# The Role of Probiotics, Prebiotics, Synbiotics, and Postbiotics in Livestock and Poultry Gut Health: A Review

**DOI:** 10.3390/metabo15070478

**Published:** 2025-07-15

**Authors:** Taojing Yue, Yanan Lu, Wenli Ding, Bowen Xu, Cai Zhang, Lei Li, Fuchun Jian, Shucheng Huang

**Affiliations:** 1 College of Veterinary Medicine, Henan Agricultural University, Zhengzhou 450046, China; ssjjkkk921@outlook.com (T.Y.); lyn13526541233@outlook.com (Y.L.); aiding3011@outlook.com (W.D.); xu18539752600@outlook.com (B.X.); 2 Henan International Joint Laboratory of Animal Welfare and Health Breeding, Henan University of Science and Technology, Luoyang 471023, China; zhangcai@haust.edu.cn; 3 College of Veterinary Medicine, Yunnan Agricultural University, Kunming 650202, China

**Keywords:** animal welfare, gut health, gut microbiota, probiotics, prebiotics, synbiotics, postbiotics

## Abstract

Background: The gut health of livestock and poultry is of utmost importance as it significantly impacts their growth performance, disease resistance, and product quality. With the increasing restrictions on antibiotic use in animal husbandry, probiotics, prebiotics, synbiotics, and postbiotics (PPSP) have emerged as promising alternatives. This review comprehensively summarizes the roles of PPSP in promoting gut health in livestock and poultry. Results: Probiotics, such as *Lactobacillus*, *Bifidobacterium*, and *Saccharomyces*, modulate the gut microbiota, enhance the gut barrier, and regulate the immune system. Prebiotics, including fructooligosaccharides, isomalto-oligosaccharides, galactooligosaccharides, and inulin, selectively stimulate the growth of beneficial bacteria and produce short-chain fatty acids, thereby improving gut health. Synbiotics, combinations of probiotics and prebiotics, have shown enhanced effects in improving gut microbiota and animal performance. Postbiotics, consisting of inanimate microorganisms and their constituents, restore the gut microbiota balance and have anti-inflammatory and antibacterial properties. Additionally, the review looks ahead to the future development of PPSP, emphasizing the importance of encapsulation technology and personalized strategies to maximize their efficacy. Conclusions: Our aim is to provide scientific insights for PPSP to improve the gut health of livestock and poultry.

## 1. Introduction

As researchers have recently explored gut diseases, gut health has become an important indicator of animal and human health. For animals, gut health determines growth performance, disease resistance, and product quality. However, prolonged use of antibiotics can exacerbate the emergence and growth of resistant bacteria, leading to an imbalance in gut microbiota and disrupting the intestinal micro-ecological environment [1]. Additionally, the accumulation or residue of antibiotics in the environment and animal products poses significant harm to human health, thus raising widespread concern nowadays [2]. Since 1986, Sweden has gradually eliminated the use of antibiotics in livestock feed, and since 2006, the European Union has also prohibited the use of antibiotics in animal feed [3]. Since 2014, the FDA has issued regulations to restrict the use of growth-promoting antibiotics in livestock. A three-year phase-out plan for prophylactic antibiotic use in animal feed was scheduled to be completed by 2017, when the ban on antibiotics for growth-promoting purposes in livestock would be fully enforced. Notably, the therapeutic use of antibiotics remains permissible, and antibiotics may be used to treat animal diseases as permitted by veterinary prescriptions [4]. From 2020, China also implements a strict ban on antibiotic. Moreover, under the current domestic breeding environment, intestinal diseases in livestock and poultry are occurring frequently. Therefore, reducing and banning antibiotics has become a trend in the development of animal husbandry, and the search for new antibiotic substitutes has become an urgent problem to solve [5]. Feed additives now play a vital role in the livestock and poultry industry as well as in the healthcare system. Growing evidence has demonstrated that feed additives can promote animal growth and production, enhance immunity and bone health, and provide health protection benefits [6,7,8,9]. Among them, probiotics and prebiotics are widely used as two emerging feed additives that can have beneficial effects on host health. According to the revised definition of the Food and Agriculture Organization (FAO)/World Health Organization (WHO), probiotics are defined as live, non-pathogenic microorganisms that, if given in sufficient doses, can contribute to the health of the host [10]. Prebiotics are a substrate selectively utilized by host microorganisms and are beneficial to health [11]. So far, the research on probiotics and prebiotics is relatively mature, and both are widely used to regulate gut microbiota (GM) to prevent and control diseases. The concepts of synbiotics and postbiotics have also received increasing attention. Synbiotics are commonly defined as intentional combinations of probiotics and prebiotics, designed to exert synergistic health benefits. Postbiotics, conversely, refer to inanimate microorganisms and/or their bioactive components, which can confer physiological advantages to the host through mechanisms independent of live microorganisms. Probiotics, prebiotics, synbiotics, and postbiotics (PPSP) are considered to be suitable gut microbiota regulators due to their high safety profile and good bioactivity and have been shown to improve the gut environment to promote animal health [12].

The gastrointestinal tract (GIT) serves as the organ with the most extensive interface with the external environment within the host. It is the principal place for digestion and absorption of nutrients in animals, and it is the material basis and guarantee for maintaining the normal host metabolism. In addition, the gut can also act as the animal’s immune organ to play the role of resistance to external germs. At the same time, the gut is also the largest micro-ecosystem, which bears the burden of secreting various physiological regulatory hormones [13]. Based on the above effects, gut health plays an essential role in the growth and development of animals and the quality of products. Therefore, maintaining gut health is necessary for the overall health of animals.

The gut is one of the mucosal surfaces with the most prominent features and in-depth research of microbial communities. Under healthy conditions, the gut microbiota composition is stable and beneficial for maintaining host health. However, the balance of GM may be disrupted in pathological situations, causing potentially harmful consequences to the host [13,14]. The GM is a complex ecosystem containing thousands of microorganisms such as bacteria, archaea, viruses, fungi, etc. These microorganisms form a complex network of interactions, which play a wide-ranging regulatory role in animal health and are also referred to as the “second genome” [15]. The GM not only serves a natural barrier to maintain the integrity of the gut epithelium and prevents invasion by pathogenic microorganisms but also acts on the gut immune system to regulate the secretion of antibodies in the gut mucosa, thereby affecting innate immunity and acquired immunity [16]. In the past decade, the GM has gradually attracted the attention of a wide range of researchers. Numerous studies have shown that some diseases can lead to changes in the composition or ratio of the GM [13,15,16,17,18,19]. Because gut microbiota is not permanent and static, it can be altered by a variety of factors such as lifestyle, environment, diet, and antibiotics. Once the GM is disordered, it will aggravate the development of various gut diseases, such as inflammatory bowel disease, primary sclerosing cholangitis, irritable bowel syndrome, and chronic constipation [18,20]. Therefore, the direct interaction of GM with various diseases is crucial. It is widely recognized that gut microbiota homeostasis is an important component of the body’s metabolic system, so one of the most direct mechanisms for improving metabolic function is to regulate the balance of beneficial and conditionally pathogenic bacteria. In addition, the GM further influences metabolic processes by continuously releasing bioactive substances, some of which may be transferred to the circulation as specific ligands. In this process, gut integrity maintains gut health by blocking the transfer of harmful bacterial metabolites [12]. In recent years, GM has been shown to play an important role in carbohydrate metabolism, neuroendocrine regulation, and immunomodulation through the gut–*X* axis, thereby improving the treatment of disease, and is therefore seen as a key target for promising new therapies [15,21]. In this review, we summarize some of the studies and mechanisms of probiotics, prebiotics, synbiotics, and postbiotics that play a role in promoting gut health by interfering with the GM, intending to shed some light on gut health in livestock and poultry.

## 2. Probiotics and Gut Health

**Figure 1:**
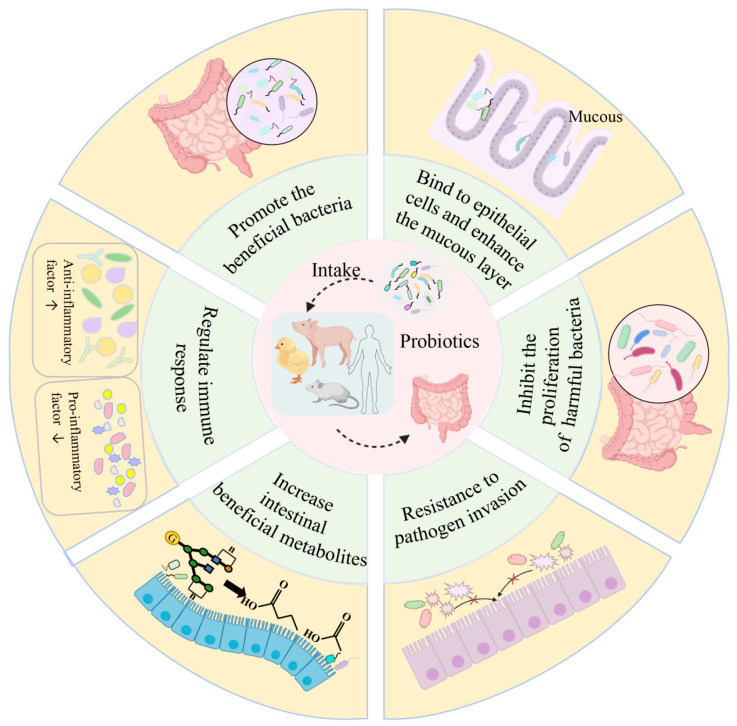
**The main mechanism by which probiotics promote intestinal health.** Probiotics improve intestinal health, and thus overall health, primarily by balancing the gut microbiota (promoting the proliferation of beneficial bacteria and inhibiting the growth of harmful bacteria), binding to epithelial cells and enhancing the mucous layer, resisting the invasion of pathogens, regulating the immune system, and increasing the production of metabolites that are beneficial to the host’s health. (This image was created with PowerPoint).

Probiotics are defined as “living microorganisms” that can bring health benefits to the host when administered in a sufficient dose [22]. In 1908, Russian Nobel Prize winner Elie Metchinkoff discovered that beneficial bacteria extracted from yogurt could regulate the GM, promoting GM stability, reducing the release of toxic compounds by pathogens into the intestines, and enhancing overall health. This discovery laid the foundation for the concept of medical probiotics [23]. Since then, probiotics have been extensively researched and applied as a new concept in many diseases [24,25]. In 1954, Ferdinand Vergin [26] first introduced the term “probiotics” in medical terminology while highlighting the negative effects of excessive use of antibiotics to the GM, demonstrating the positive impacts of probiotics on human health and the importance of their development. To date, probiotics have been demonstrated to have great potential for treating a variety of diseases by modifying the GM, primarily gastrointestinal disorders (including acute infectious diarrhea, antibiotic-associated diarrhea, *clostridium difficile*-associated diarrhea, ulcerative colitis, and functional gastrointestinal disorders). Furthermore, they can improve the management of some metabolic diseases and other extraintestinal diseases, such as hepatic encephalopathy [25,27]. Numerous studies conducted since the early 20th century have demonstrated that human and animals can achieve therapeutic effects on diseases by ingesting probiotic foods containing specific bacterial strains. This has led researchers to further explore the development of probiotic foods and the regulation of GM [24,28,29]. Candidates for microbial probiotics generally include four primary characteristics: the ability to survive in the target organ, interaction with the host system, safety, and disease resistance. Most probiotics are taken orally from the mouth into the GIT to reach the target organ. This suggests that screening for potential probiotic strains must include resistance to the internal environment of the GIT (e.g., low pH, digestive enzymes, and body temperature), the ability to colonize mucosal surfaces, and maintenance of the stability of the GM (Figure 1). Meanwhile, the selected strains are not pathogenic, toxin-producing, or threatening to host health [30]. Microorganisms used as probiotics come from different genera and species. According to the current research, probiotics mainly include yeasts and bacteria (such as *Lactobacillus*, *Lactococcus*, *Leucococcus*, *Diplococcus*, and *Bifidobacterium*) [31,32]. The most studied probiotics include *Lactobacillus*, *Bifidobacterium*, and *Saccharomyces*. Some of the strains often used as probiotics are listed in Table 1.

### 2.1. Lactobacillus

Members of the *Lactobacillus* family have long been recognized as one of the most abundant microbiota in the GIT, and their presence has been linked to the maintenance of gut health. *Lactobacillus*, a Gram-positive bacterium, is considered a very important probiotic. Now, it is widely used in human and animals to treat and prevent a wide range of diseases, especially in gastrointestinal disorders [49,50]. *Lactobacilli* can adhere to the epithelial cell layer of the gastrointestinal system to exert a protective effect on the gut and show an adequate therapeutic effect in gut disorders. First, *Lactobacillus* binds to the surface of the host’s epithelial cells, reducing the harmful effects of bacterial enteric pathogens [51]. Second, *Lactobacilli* can induce gut epithelial cells to produce and secrete mucin, increase the mucus layer around the gut, enhance the protective effect on the gut, and remove gut pathogens [52]. A study by Johnson-Henry et al. concluded that probiotic *Lactobacillus* strains blocked *Escherichia coli (E. coli)* adhesion through the epithelial barrier, which suggests that probiotic *Lactobacillus* exert an enteroprotective effect by preventing pathogenic bacteria from adhering to the gastrointestinal epithelium [33]. Yang et al. [53] supplemented piglets with *Lactobacillus* and found that it was possible to increase the abundance and number of *Lactobacillus* and other indigenous probiotics to help create an optimized microbial community, which in turn improved growth and immunity in piglets. It also promotes the production of prebiotics, which promotes piglet growth and reduces the risk of pathogen infection, maximizing host feed utilization. Zhang et al. [54] supplemented yaks with *Lactobacillus brevis* and found that it enhanced the expression of intestinal mucus and tight junction proteins, thereby treating *E. coli* in yaks. Similarly, the research conducted by Tellez et al. [55] found that administering an appropriate amount of *Lactobacilli* to broiler significantly reduces *Salmonella*. *Lactobacillus rhamnosus* GG (LGG) belongs to a group of probiotic *Lactobacillus* spp., originally isolated from healthy human feces by Barry R. Goldin and Sherwood L. Gorbach in 1983 [56]. Studies have shown that LGG has powerful adhesion properties, which can remove or reduce the residence of pathogenic bacteria in the organism, produce substances that can antagonize food-borne pathogenic bacteria, and also directly participate in the repair of damage to the gut mucosal barrier function and gut mucosal immunomodulation of the host, ultimately maintaining gut health [34,56,57,58]. Zhang et al. [59] found that feeding LGG to pre-weaned calves increased their growth performance and improved rumen fermentation to increase propionic and butyric acid concentrations normally. It also diversified the composition of the bacterial community in the rumen and regulated the balance of GM in the rumen, promoting early development and weaning of newborn calves. On the other hand, the immunomodulatory effect of *Lactobacillus* has a vital role in the maintenance of gut health. Treg cells are capable of secreting anti-inflammatory cytokines such as IL-10 and IL-17, which are essential for the maintenance of immune homeostasis. *Lactobacillus* can restore gut homeostasis and further contribute to the amelioration of inflammatory diseases. In a recent study, LGG was shown to increase sIgA concentration, attenuate serum IL-6 elevation, and modulate GM to ameliorate diarrhea induced by *E. coli* K88 in post-weaning piglets [60]. Moreover, Chen et al. conducted a study on broilers infected with *Salmonella Typhimurium* and found that administering a probiotic containing lactic acid bacteria to the affected chickens reduced the expression of IL-6, IL-1β, and IFN-γ while increasing the expression level of IL-10 in the cecal tonsils, thereby alleviating the progression of the disease [35]. There is also research indicating that administering a certain amount of lactic acid bacteria, such as *Lactobacillus acidophilus*, *Lactobacillus salivarius*, and *Lactobacillus plantarum*, orally to commercial broilers on a weekly basis can enhance the antibody levels and cell-mediated immunity within the chickens. Another study on weaned piglets reported an increase in fecal *Lactobacilli* in weaned piglets fed *Lactobacillus plantarum* JDFM LP11, and macrogenomic analysis showed a higher relative abundance of colony diversity and richness [36]. In conclusion, *Lactobacillus* has been used in both human and animal husbandry for its probiotic effects to ameliorate gut diseases.

### 2.2. Bifidobacteria

*Bifidobacteria* is a Gram-positive bacterium and the dominant microbiota in both human and animals guts that is a Generally Recognized as Safe (GRAS) probiotic [61]. *Bifidobacteria* are the dominant microbiota in the gut of humans and animals, and their abundance is related to factors such as age, health status of the organism, and feeding practices. In young animals, the abundance of bifidobacteria is high and decreases with age. Firstly, *Bifidobacteria* can withstand adverse factors in the GIT, ultimately adhering to intestinal epithelial cells to provide a defense against harmful bacterial invasion. A study by Farooq et al. [62] demonstrated that dietary supplementation with *Aeriscardovia aeriphila*, which is known as *Bifidobacterium aerophilum*, in broiler chickens significantly increased the mRNA expression levels of IL-10 (*p* < 0.05) and IL-4 (*p* < 0.001) in the intestinal mucosa. Concomitantly, the abundance of *Phascolarctobacterium* and *Barnesiella* was increased. These changes collectively inhibited harmful bacterial colonization, promoted the production of beneficial metabolites (e.g., short-chain fatty acids (SCFAs)), facilitated intestinal barrier repair, restored intestinal functionality, and safeguarded gut health. A recent study reported that, compared to broilers that receive single probiotic or no probiotic supplementation, those administered with a complex probiotic containing a high abundance of *Bifidobacterium* exhibit superior slaughter performance. This phenomenon may be attributed to the resilient strains of *Bifidobacterium* in the GIT, which effectively inhibit the colonization of pathogenic bacteria and stimulate the development of intestinal structures, thereby optimizing the absorption and utilization of nutritional components from feed [63]. Additionally, a different study revealed that *Bifidobacteria* are essential for the metabolism of indigestible oligosaccharides, which can be metabolized into lactic and acetic acid, improving the environment of GM and fecal viscosity, contributing to alleviating gut diseases [64]. In addition, *Bifidobacteria* can increase the proportion of beneficial bacteria in the GM through cross-feeding. According to Turroni et al. [65], co-culturing of *Bifidobacterium bifidum* and *Bifidobacterium shortum* in vivo led to a significant enhancement in the metabolism of the GM and an increase in SCFAs production, which was mainly due to the provision of nutrients for the growth of butyric acid-producing probiotic bacteria through cross-feeding.

Notably, the majority of gastrointestinal disorders impair GM homeostasis and weaken the immune system, resulting in disruption of the immune system. *Bifidobacteria* supplements are crucial because of their immune-boosting properties,, which help to improve gastrointestinal problems and restore GM homeostasis. *Bifidobacteria* have been reported to alter dendritic cell function and modulate gut immune homeostasis to initiate protective measures against pathogens. Zhi et al. [66] fed broilers moderate amounts of *B. laterosporus* S62-9, which resulted in a 7.2% increase in body weight and promoted maturation of immune organs and increased serum immunoglobulin concentrations in the S62-9 group compared to the control group. In addition, S62-9 supplementation increased the relative abundance of beneficial bacteria, including *Akkermansia*, *Bifidobacterium*, and *Lactobacillus*, while decreasing the relative abundance of pathogens, including *Klebsiella* and *Pseudomonas*. In another study, supplementation with *Bifidobacterium shortum* MCC-117 was effective in modulating the inflammatory response triggered by enterotoxin-producing *E. coli* (ETEC) in porcine gut epithelial cells, stimulated the expression of Toll-like receptor 2 (TLR-2) and cyclooxygenase-2 (COX-2) in the ileal epithelium, and blocked cytokine-induced apoptosis, thereby reducing mucosal damage and maintaining gut integrity. Zhang et al. [67] administered a composite probiotic at a dosage of 10 g/kg (comprising *Lactobacillus*: *Lactococcus*: *Bifidobacterium* in a ratio of 1:1:2) to broiler chickens, and the results from 16S rRNA amplicon sequencing indicated that the probiotics significantly increased the relative abundance of _*Bacteroidales*, _*Rikenellaceae*, and _*Alistipes* in the broilers, while also enhancing the cecal microbiota of the broilers at 42 days of age. Furthermore, the addition of probiotics resulted in a decrease in the relative abundance of harmful microorganisms. Therefore, as a major probiotic in the gut, *Bifidobacteria* mainly exerts its probiotic effect by preventing the invasion of pathogenic bacteria, promoting the growth of beneficial bacteria, and regulating the immune system.

### 2.3. Saccharomyces

Nowadays, a great deal of research is focused on the exploitation of bacteria-derived probiotics, but research into yeast-derived probiotics has been relatively limited. One of the drawbacks of probiotics of bacterial origin that has come to the fore is their ability to transfer resistance genes to pathogenic bacteria. In comparison to bacterial probiotics, *Saccharomyces* offer several advantages: exhibiting greater resistance to gastrointestinal enzymes and bile salts, as well as variations in pH and temperature. These ensures that yeast probiotics reach the intestines largely intact. *Saccharomyces* possess intrinsic resistance to antibiotics and enhance the host’s immune response, which accounts for some of the positive health benefits attributed to *Saccharomyces* [68]. In recent years, there has been a growing recognition that fungal probiotics can influence the gut as well as other organs in humans and animals by triggering adaptive immune responses and local mucosal protective mechanisms. Compared to bacterial probiotics, the potential of *Saccharomyces* as probiotics remains insufficiently explored and warrants greater attention [69]. According to previous studies, several strains of the yeast genera such as *Saccharomyces*, *Pichia*, *Kluyveromyces*, *Candida*, and *Schizosaccharomyces* have been shown to have potential probiotic properties [70]. Among them, *Saccharomyces* are the most researched and common probiotic yeasts, and *Saccharomyces boulardii*, a member of the *Saccharomyces cerevisiae* family, has been demonstrated to show great promise in improving gut health. Moreover, *Saccharomyces boulardii* CNCM I-745 is the first yeast probiotic for the treatment of human clinical diseases [71]. Compared to other bacterial probiotics, it has a broader temperature threshold and reacts more slowly to pH changes, particularly those that occur in acidic conditions. Secondly, it does not cause antibiotic resistance but has a therapeutic restorative effect on infections caused by harmful pathogens such as *Clostridium difficile*, *Salmonella*, *E. coli*, viruses, and *Candida albicans* [71,72]. It is well known that probiotics generate antimicrobial substances that either prevent [73] or encourage [74] the development of gut microbes. Several studies have demonstrated that *Saccharomyces boulardii* intervention can prevent inflammation by promoting immune function, increasing SCFAs production and *Bacteroidetes* abundance, and reducing the abundance of *Firmicutes* and *Proteobacteria*, thereby improving gut barrier and preserving gut health [72,75,76].

*Saccharomyces cerevisiae* var. *boulardii* is a kind of *Saccharomyces boulardii* that many studies have focused on as a kind of yeast probiotic. The attack of pathogenic microorganisms on the host GM leads to a dysregulation of its homeostasis and reduces the total probiotic load in the gastrointestinal system, thus causing inflammation and secondary infections [77]. Based on previous studies, *Saccharomyces cerevisiae* var. *boulardii* was found to enhance defense against different gut pathogens. The main effect is that its cell wall can bind gut pathogens and reduce their reproduction and growth by accelerating bacterial excretion through feces, thus preventing bacterial adhesion and translocation in the gut epithelial cells [78]. Gedek [79] discovered that the cell wall of *Saccharomyces cerevisiae* var. *boulardii* exhibits binding capacity to *E. coli* and *Salmonella* spp. In addition, yeast cells or cell wall particles can modify the surface receptors of *C. difficile* adhesion depending on their proteolytic activity and spatial hindrance. A study demonstrated that the pretreatment of *Clostridium difficile* or *Vero cells* with *Saccharomyces cerevisiae* var. *boulardii* or its cell wall particles decreases bacterial adhesion to *Vero* cells [80]. Lessard et al. [38] also found that giving *Saccharomyces cerevisiae* var. *boulardii* to pigs reduced the adhesion of enterotoxigenic *E. coli* to mesenteric lymph nodes. *Saccharomyces boulardii* not only adheres to and inhibits the activity of toxins released by *Vibrio cholerae* but also inhibits cyclic adenosine-induced chloride monophosphate secretion, significantly reducing the mobility of sodium and chloride produced by *Vibrio cholerae*. In conclusion, probiotic yeasts can be directly therapeutic for *C. difficile* disease by targeting toxins and receptors [69]. In addition, Bermudez-Brito et al. [81] found that continuous administration of *Saccharomyces cerevisiae* var. *boulardii* for several weeks alleviated the disease process and was ultimately able to stabilize the host gut microenvironment by allowing the host to heal. Brousseau et al. [39] investigated the effects of feeding *Saccharomyces cerevisiae* subsp. *boulardii* to lactating sows and their piglets, assessing its regulation of the ileal and colonic microbiota after 37 days of feeding. The results indicated a significant impact of *Saccharomyces cerevisiae* subsp. *boulardii* on the establishment of *Porphyromonadaceae* and *Ruminococcaceae* in the colon compared to the control group, which demonstrates that supplementation with *Saccharomyces cerevisiae* subsp. *boulardii* can influence the intestinal and colonic microbiota of weaned piglets in a strain-dependent manner, suggesting its potential as a feed additive to modulate bacterial populations associated with gut health. A recent study examining fecal samples from pre-weaned calves found that the microbiota of healthy and diarrheic calves differed at 6 and 8 weeks of the trial. However, supplementation with *Saccharomyces cerevisiae* var. *boulardii* demonstrates the potential to modulate the microbiota composition of diarrheic heifers, restoring it to levels analogous to those in healthy heifers. Consequently, this intervention improves the calf’s gut health [82]. In poultry applications, studies have demonstrated that continuous feeding of 600 mg/kg *Saccharomyces cerevisiae* to Pekin ducks for 42 consecutive days increased intestinal barrier-associated mRNA levels of claudin3, occludin, inBP, ZO-1, and MUC2. Concurrently, this intervention enhances the abundance of *Bacteroidetes* and *Ruminococcaceae_UCG-014*, thereby promoting gut health [83]. Another investigation in broilers supplemented with *Saccharomyces boulardii* revealed increased gut *Lactobacilli* abundance and microbial diversity, which improves food digestion, nutrient absorption, and growth performance [84]. Thus, these studies highlight the therapeutic benefits and translational potential of yeasts as probiotics for human and animal health. However, further exploration across diverse species remains critical. Notably, *Saccharomyces* species may exhibit antimicrobial resistance attributed to inherent differences in cell wall structure and ribosomal composition or develop tolerance to antifungal agents through drug exposure, leading to a possible reduction in their therapeutic efficacy. Future research should therefore address these limitations through targeted yeast modifications to optimize their application in agricultural production.

## 3. Prebiotics and Gut Health

**Figure 2:**
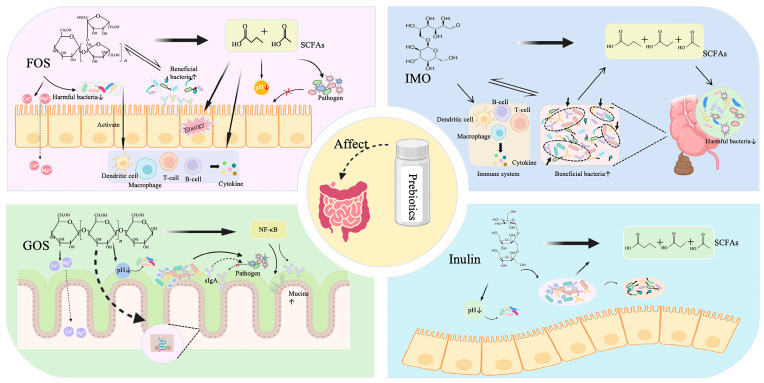
**The role of different prebiotics on intestinal health.** Different prebiotics generally promote the growth of beneficial bacteria, especially *Bifidobacterium* and *Lactobacillus*, but also can be broken down by beneficial bacteria into short-chain fatty acids, which promote each other with probiotics and provide energy for epithelial cells. In addition, they inhibit the proliferation of harmful bacteria and impede the invasion of pathogens into the gut. Prebiotics also regulate the immune system and promotes immune response, while promoting mineral absorption and strengthening the intestinal barrier. (This image was created with PowerPoint).

Prebiotics were first defined by Gibson and Roberfroid as “non-digestible food components that improve host health by selectively stimulating the growth and/or activity of one or a limited number of bacteria in the colon, thereby having a beneficial effect on the host” [85]. With advances in 16S rRNA microbiome sequencing of gastrointestinal microbial communities, researchers have gained new insights into both prebiotic-related properties and compounds that qualify as prebiotics [86,87]. In 2016, a panel of experts from the International Society for the Science of Probiotics and Prebiotics (ISAPP) reviewed the definition of prebiotics and defined them more broadly as “substrates that can be selectively utilized by host microbiota to confer health benefits” [88]. Nowadays, prebiotics are substances that stimulate the growth of microorganisms, whose composition does not include bacteria, and which are commonly used to maintain a normal GM and restore homeostasis to the internal environment [89,90,91,92]. Consumption of prebiotics restores the symbiotic relationship between the microbiota and the host, providing health benefits to the host [93]. Prebiotics are widely found in some plants such as soy, oats, and asparagus. Probiotics are designed to balance the GM by stimulating the growth of beneficial bacteria in the gut (such as *Lactobacillus* and *Bifidobacterium*) and promote gut metabolic activity to produce a series of metabolites that are beneficial to the host and ultimately achieve the goal of maintaining gut health (Figure 2) [21,89,90]. Prebiotics are usually composed of 2–20 monomers (including glucose, galactose, fructose, and/or xylose) that are not usually hydrolyzed in the gut. In addition, prebiotics have an indigestion in the colon that contributes energy due to their caloric value and participation in fermentation [94]. Among the many prebiotics, fructooligosaccharides (FOS), isomalto-oligosaccharides (IMO), galactooligosaccharides (GOS), and inulin have been widely studied.

### 3.1. Fructooligosaccharides

Fructooligosaccharides (FOS), an oligosaccharide fiber, is naturally found in plants such as chicory, onions, leeks, and asparagus. However, studies have also found that yacon has the highest concentration of FOS [95,96]. FOS cannot be digested in the small intestine but is selectively used as a source of nutrients mainly by bacteria in the colon (such as *Bifidobacteria*) [97,98]. Based on previous research, FOS has attracted attention for its functional properties and economic potential [99,100]. FOS selectively stimulates the growth of *Bifidobacteria* and *Lactobacilli* in the colon because of their interactions and is degraded by these colon-specific anaerobic bacteria to produce different SCFAs. As a result, SCFAs not only lead to a decrease in colonic pH but serve as an energy source for host epithelial cells, modulate the gut immune response, enhance gut barrier function, and inhibit the adhesion of pathogenic bacteria [101].

As a functional food, it has been demonstrated that regular intake of FOS can strengthen the host resistance to gut or extra-gut pathogens, promote colonic microbiota homeostasis, and increase mineral absorption (Ca^2+^ and Mg^2+^) in the GIT and thus improve host health [102,103,104]. In a study of sows and their weaned piglets, FOS supplementation was found to reduce the incidence of stillbirths and disabled piglets, increase the abundance of *Akkermansia muciniphila*, decrease the abundance of *E. coli*, and increase fecal isovaleric acid content. Meanwhile, dietary supplementation of weaned piglets with FOSs reduced diarrhea and increased short-chain fatty acid content in feces, suggesting that supplementation with FOSs can influence the composition of the host’s GM and thus exert a probiotic effect [105]. In addition, Luo et al. [106] induced enterotoxigenic *E. coli* (ETEC) in weaned piglets and treated them with FOSs, and they found that the presence of FOSs was able to reduce inflammatory cytokines in the intestinal mucosa of the ETEC pigs and improve the antioxidant capacity and intestinal barrier integrity in ETEC pigs, suggesting that FOSs have a possible probiotic and protective effect on the maintenance of intestinal epithelial function in the presence of pathogen attacks. At present, the transmission of foodborne pathogens during the growing process in poultry production remains a major problem in the poultry industry [107,108]. Based on previous studies, the addition of feed additives that target the GM and prevent the initial colonization of foodborne pathogens may help reduce overall infections [23,108,109]. A study by Ricke et al. [110] found that FOS was able to bind to high-fiber material in the gut and alter the fermentation and cecal microbial composition of the cecum in adult laying hens, thereby preventing *Streptococcus enteritidis* colonization and infection in susceptible birds. The above demonstrated the role of FOS in altering the composition of the gut microbiota by targeting specific groups of bacteria, ultimately promoting host gut health.

### 3.2. Isomalto-Oligosaccharides

Isomalto-oligosaccharides (IMO), a mixture of oligosaccharides with α (1,6) and α (1,4) glycosidic bonds [111,112], are a food additive with the advantages of low caloric value, low sweetness, and non-toxicity and can be used as a substrate for *Bifidobacterium* and *Bacteroides* [113]. Moreover, consumption of IMO can stimulate the growth of beneficial microbes (mainly *Lactobacillus* and *Bifidobacterium*) in GIT and regulate local or systemic immune responses, thus enhancing the host resistance to disease. A study by Ketabi and Dieleman [114] pointed out that *Lactobacillus* was one of the major microbes in rat fecal samples, and its abundance and total number of GM were increased in the IMO-fed rats compared to the normal- and inulin-diet rats. In addition, IMO has been reported to enrich *Bifidobacteria* while reducing *Salmonella* colonization in the cecum of broilers, thereby reducing the incidence and spread of gut diseases in broilers [115]. Wu et al. [116] and Wang et al. [117] demonstrated that IMO supplementation not only improves growth performance and enhances immune function in piglets but also increases the regulation of the abundance of beneficial bacteria (such as *Streptococcaceae* and *Collinsella*) in weaned piglets, thereby modulating the cecum GM. In addition, IMO improves constipation similarly to other fibers. According to a previous study, older adults who took an IMO-rich diet for 4–8 weeks to treat constipation had more frequent spontaneous bowel movements, and the fecal wet weight increased by 24%. At the same time, the abundance of beneficial bacteria such as *Lactobacillus*, *Bifidobacterium*, and *Enterobacteriaceae* increased, and the number of *Clostridium* decreased in the feces [118]. Tarabees et al. [119] conducted a study in which 101-day-old Cobb chicks were fed a combination of probiotics and individual microorganisms (IMO) to investigate their therapeutic effects against avian pathogenic *E. coli* (APEC) O78. The final results indicated that compared to the control group, the groups receiving the combined probiotics and IMO showed significantly increased total lactic acid bacteria and populations of *Lactobacillus* and *Enterococcus* in the broilers while also improving their growth performance, thereby protecting the broilers against the APEC O78 challenge. Pi et al. [120] supplemented 25-day weaned piglets with IMOs and found that they significantly up-regulated the expression of the anti-inflammatory cytokine TGF-β in the mucosa of the jejunum of piglets, significantly increased *Bifidobacteria* in the cecum, and significantly reduced the number of potential enteropathogenic *E. coli* and *Clostridium* species in the ileal contents. These results suggest that the application of IMO can favorably modulate the GM composition and promote gut health in piglets. Due to the prebiotic properties of IMOs, they can be utilized by some commensal bacteria in the gut to produce SCFAs such as acetate, propionate, and butyrate. Conversely, decreased production of SCFAs may further weaken the action of pathogen in the organism [121]. IMOs have been reported to increase the abundance of *Bifidobacteria* and *Lactobacilli* and butyrate production during in vitro fermentation with human fecal microbiota [119]. IMO has been a well-established functional food in Asia for decades. The ability of IMO to benefit the GM has been reported in rats, pigs, and humans, and different prebiotic potentials have been observed in several studies compared to other types of prebiotics [122,123,124,125,126]. In summary, the prebiotic potential of IMO has been proven, and their great contribution to the promotion of human and animal health cannot be ignored.

### 3.3. Galactooligosaccharides

Galactooligosaccharides (GOS) is an important food-grade oligosaccharide that cannot be digested by the human body. GOS has 1–5 galactose monomeric oligosaccharides with terminal glucose residues, which are bound by three different glycosidic bonds of Galβ (1 → 3), Galβ (1 → 6), and Galβ (1 → 4) [89,127]. As a prebiotic, GOS selectively increases the growth of beneficial bacteria in the GM, such as *Bifidobacteria* and *Lactobacilli* [128,129]. Adhesion and proliferation of beneficial microbiota leads to reduced resistance of pathogens to colonization and reduces the likelihood of pathogens disrupting normal gut homeostasis. In addition, the intake of GOS lowers the pH of the colon, which hinders the growth of pathogenic bacteria and reduces the likelihood of colon infections [130]. It also promotes the absorption of various minerals, controls lipid and cholesterol levels, and improves bowel movements and constipation [128,130,131,132]. It is beneficial to overall health. All have benefits for the overall health of the organism. Physical, chemical, immunologic, and microbial components of the gut interface are distinct from the strict requirements of the internal and external environments [133]. Of these, the physical barrier is the first line of defense against pathogen invasion and is critical to gut health [134]. The gut barrier consists mainly of epithelial cells and their neighboring intercellular junctions, such as tight junctions and subapical junctions. In addition, the integrity of the gut mucosal surface is maintained by the mucus layer, and SIgA can bind to the mucus. The dual combination of biochemical and immune barriers inhibits pathogens from adhering to, colonizing, or invading the mucosal surface, thus ensuring the stability and health of the gut internal environment [135]. It is found that GOS not only improved the GM composition of LPS-affected mice but also increased the abundance of beneficial bacteria such as *Akkermansia*, *Lactobacillus*, and *Bifidobacterium* and decreased the abundance of *Aspergillus* phylum and *Escherichia-Shigella* spp. (mucosal inflammation-associated microbiota), thereby maintaining the gut microcosmic homeostasis [136,137]. Lee et al. [138] fed pregnant sows with 30 g/d of GOS and collected colostrum 24 h postpartum to test for rotavirus-specific antibodies. Fecal samples were collected from sows and piglets three days postpartum to evaluate the presence of rotavirus A and analyze microbial composition. The results indicated that supplementation of GOS during pregnancy significantly increased the levels of rotavirus-specific IgG and IgA in colostrum compared to the control group, while also inhibiting the presence of potential pathogenic bacteria in both lactating sows and newborn piglets. At the same time, GOS therapy can also restore the integrity of the villi of the jejunum and ileum, and it has been shown that GOS can increase the secretion of mucin and SIgA through the NF-κB pathway, reduce the inflammatory response caused by LPS, and maintain the gut mucosal barrier, protecting gut health [135]. Alizadeh et al. [139] established a neonatal pig model to investigate the multifaceted effects of early dietary GOS. The results indicated that fermentation of GOS in the colon leads to a decrease in pH and an increase in butyrate levels in cecal digestive fluid. Additionally, by day 26, the populations of *Lactobacillus* and *Bifidobacterium* had increased. Furthermore, the addition of GOS was found to upregulate the mRNA expression of various tight junction proteins in the intestines of the piglets, thereby promoting a balanced GM and enhancing intestinal structure. A study of GOS to improve ulcerative colitis (UC) also showed that GOS intake reduced the abundance of *Firmicutes*, *Anaerobes*, and *Oscillospira* and increased fecal volume in patients with UC [140]. In conclusion, all of the above studies have demonstrated the probiotic properties of GOS and the important role it plays in maintaining gut health as a potential substance for future functional foods and animal feed additives.

### 3.4. Inulin

Inulin is a soluble dietary fiber that is widely found as a reserve polysaccharide in a variety of plants, and its main sources include Jerusalem artichoke, chicory, barley, and garlic [141]. Inulin is a hard-to-digest carbohydrate with varying degrees of polymerization (DP), usually consisting of fructose units (2–60 units) and terminal glucose units. Chemically, inulin is a fructan formed from D-fructofuranose molecules linked by β-(2,1)-glycosidic bonds, which are usually bound to glucose residues at the end [142]. In 1804, the German scientist Valentine Rose discovered inulin in the roots of Elecampane, which was named inulin by Thomson in 1817 [143]. Inulin has a variety of biological functions such as prebiotic effect, antioxidant, and immunomodulation. [143,144,145]. It is widely believed that inulin regulates the GM by stimulating the growth of beneficial bacteria, thus exerting its prebiotic effect [141]. *Lactobacillus* and *Bifidobacterium* are generally considered to be the most commonly studied beneficial bacteria for host health. The special β-(2,1) configuration of the heterodimer C2 of inulin leads to the fact that it is hardly hydrolyzed by digestive enzymes in the gut but it can be decomposed by microorganisms, and at the same time, it can be utilized by *Lactobacillus* and *Bifidobacterium*, which promotes the proliferation of beneficial bacteria and regulates the balance of the GM [146]. In the gut, inulin fermentation leads to changes in gut microbial composition or the relative abundance of different bacterial species. Usually, intaking inulin can increase the number of *Bifidobacteria* and *Lactobacilli* and other beneficial bacteria [147]. Li et al. [148] supplemented lactating piglets with inulin and sampled them three weeks after weaning and found that the highest chorionic crypt ratio in the jejunum, as well as increased concentrations of propionic acid, isobutyric acid, or total short-chain fatty acids in the cecum and colon, and a decrease in relative abundance of *E. coli* and *Enterobacteriaceae* in the colon, improved the intestinal development of lactating piglets in IN-0.5-treated piglets. A study found that the addition of oligofructose (with a mean DP of 4) and inulin (with a mean DP of 10) to mouse diets resulted in a significant increase in the abundance of *Bifidobacteria* and *Lactobacilli* and a decrease in the abundance of the *Aspergillus* phylum, which contains most of the deleterious bacteria, compared with controls [149]. In addition, inulin can reduce gut pH and promote the production of SCFAs during the fermentation of GM [150]. Nabizadeh et al. administered inulin at a dosage of 10 g per kilogram of feed to broiler chickens and observed that the addition of inulin led to a reduction in *E. coli* counts, a decrease in cecal pH levels, and a significant increase in the number of *Bifidobacteria*. The GM is highly sensitive to pH changes, which can typically result in alterations in bacterial composition, such as inhibiting the proliferation of pathogenic *Salmonella* or *E. coli*. Additionally, Rebolé et al. found that the inclusion of inulin in the feed resulted in an increase in *Lactobacilli* counts in the ceca of broiler chickens. Moreover, Le Bastard et al. [147] indicated that inulin supplementation decreased the relative abundance of *Bacteroides*, which may be related to the decrease in gut pH. Meanwhile, the levels of *Bifidobacterium*, *Anaerobacter*, *Enterococcus faecalis*, and *Lactobacillus* were significantly increased. In addition, there was an increase in the abundance of *Ruminococcus*, *Phascolarctobacterium*, *Akkermansia*, *Blautia*, and *Lachnospiraceae*, which are bacteria associated with the production of SCFAs [147,151,152]. In summary, the prebiotic effect of inulin is mainly realized by promoting the growth of beneficial bacteria and at the same time interacting with the GM to produce some metabolites to achieve the protective and stabilizing effect on the gut.

## 4. Synbiotics and Gut Health

**Figure 3:**
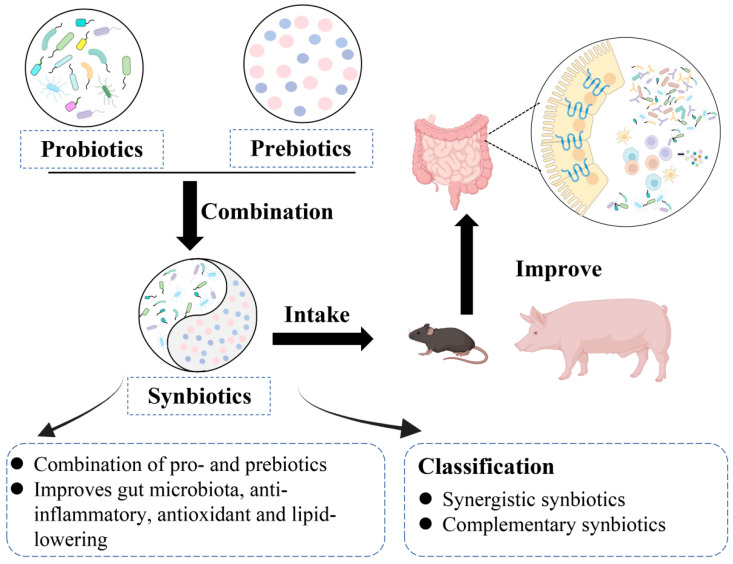
**Composition and physiological function of synbiotics.** Synbiotics are combination of probiotics and prebiotics (circles of different colors represent different prebiotics)., which can be categorized into complementary and synergistic formulations. When livestock or poultry intakes synbiotics, it can enhance gut health by improving the gut microbiota and intestinal barrier, thereby boosting animal production performance and promoting host health. (This image was created with PowerPoint).

Synbiotics, also known as synbiotics, are a mixture of probiotics and prebiotics, which are mixed in different proportions according to need [153]. Synbiotics have been shown in several studies to improve GM, anti-inflammatory, antioxidant, and lipid-lowering biological functions [154,155,156] (Figure 3). The ways in which synbiotics enhance the effects of probiotics and prebiotics can be divided into complementary synbiotics and synergistic synbiotics [157]. Complementary synbiotics are a mixture of probiotics and prebiotics that have their own respective beneficial effects and which independently exhibit one or more of these beneficial effects. However, the components of complementary symbiotics must meet minimum requirements (such as specified content, appropriate live bacteria count, and adequate evidence of beneficial effects on health) [158]. Oh et al. [159] treated azomethane/dextrose sodium sulfate-induced colitis-associated colorectal cancer mice with a synbiotic preparation combining *Lactobacillus gasseri* and a novel prebiotic, trichothecene leaf extract, which was able to reduce *Staphylococci* and increase the number of *Lactobacillus*, *Bifidobacterium*, and *Akkermansia* in the gut of mice, as well as increase the production of SCFAs. In addition, the study demonstrated that the synbiotic was effective in reducing inflammation and carcinogenesis induced by gut injury in model mice and helped to restore the balance of GM, but the prebiotic did not selectively stimulate *Lactobacillus gasseri* [159]. Prebiotics are primarily saccharides of varying degrees of polymerization, structure, composition, and source, and probiotics vary in their ability to metabolize saccharides. Synergistic synbiotics are combinations of probiotics with prebiotics that they can specifically metabolize, and the selective stimulation of the probiotics by the prebiotics in the combination is known as synergism. In contrast to complementary synbiotics, rationally designed synergistic synbiotics do not need to meet the minimum requirements for probiotics and prebiotics [160]. Morshedi et al. [161] found that treating diabetic rats with a synbiotic formulation of *Lactobacillus plantarum* and inulin, with *Lactobacillus plantarum* utilizing inulin as a substrate, as compared to inulin or *Lactobacillus. plantarum* alone was able to increase the number of *Lactobacillus delbrueckii* and *Lactobacillus acidophilus* and decrease the number of *Clostridium*, improve GM disorders and gut dysplasia in diabetic rats, and significantly enhance the cognitive function of rats. The results demonstrated that the simultaneous intake of *Lactobacillus plantarum* and inulin had a synergistic effect. However, in the current clinical and commercial use of synbiotics, most are complementary synbiotics. Therefore, it is crucial to explore rational combinations of probiotics and their corresponding prebiotics in synbiotic formulations so as to better utilize their biological effects. In a study using a synbiotic mixture of probiotics such as *Lactobacillus acidophilus*, *Bifidobacterium lactis*, *Bifidobacterium longum*, and *Bifidobacterium* in combination with galactose in treating an obese population, it was noted that synbiotic supplementation increased the abundance of health-positive gut bacteria, particularly *Bifidobacterium* and *Lactobacillus lactis*, as well as increasing the abundance of the GM, correcting the disruption of the GM caused by obesity [154].

In addition, studies have shown that the addition of synbiotics to animal diets can improve animal performance, increase the activity of gastrointestinal digestive enzymes, promote the colonization of beneficial bacteria, inhibit the proliferation of harmful bacteria, and balance the composition of their GM. At the same time, it can increase the gut mucosal barrier function, reduce gut permeability, and improve the morphology of the small intestine [155]. Du et al. [162] fed a synbiotic consisting of *Clostridium butyricum*, *Bacillus licheniformis*, *Bacillus subtilis*, yeast, and xylooligosaccharide to heat-stressed broilers and found that, compared to the heat-stressed group without synbiotic supplementation, synbiotic supplementation increased broiler body weight, decreased serum IL-1β levels, and reversed the changes in the mRNA expression levels of jejunal IL-1β and ZO-1, suggesting that the synbiotic can be used as a dietary supplement to improve the growth performance, immune function, and intestinal barrier function of broilers subjected to heat stress. Furthermore, studies have shown that feeding piglets with dietary supplements containing complex probiotics (*Lactobacillus plantarum* B90 and *Saccharomyces cerevisiae* P11) or synthetic biotics (complex probiotics + xylo-oligosaccharide) can improve piglet survival and lipid metabolism by altering the diversity and composition of GM [163]. In recent years, studies on improving the next generation by feeding synbiotics to lactating female livestock have gradually been confirmed. Ma et al. [164] fed sows during pregnancy and lactation with a synbiotic preparation of *Plantactobacillus B90*, *Saccharomyces cerevisiae* P11, and xylo-oligosaccharide (XOS) to study the impact of their milk on piglets. Detecting pig-related indicators found that the supplement of the synbiotics significantly increased the abundance of *Anaerovorax*, *Sharpea*, and *Butyricicoccus*, as well as promoting the expression of mRNAs such as E-cadherin, Occludin, ZO-1, ZO-2, IL-10, and INF-α. Moreover, there is a positive correlation between colon-oxygenal bacteria and acetic acid and SCFAs levels. These results demonstrate that the addition of synbiotic preparations to the diet of sows can improve nutrient metabolism and intestinal barrier permeability, reduce oxidative stress, and affect the composition and metabolic activity of the colonic microbiota in suckling piglets. In conclusion, synbiotics can be applied to humans or animals to improve gut health by targeting GM while also improving various diseases.

## 5. Postbiotics and Gut Health

With the rise of public health awareness, foods or supplements with health functions have been gradually favored by consumers. Numerous studies have confirmed that microorganisms are crucial for promoting the health of human or animal organisms. Probiotics, as beneficial microorganisms with the ability to regulate GM and ameliorate diseases, have been attracting the attention of researchers in the fields of food, feed, and medicine, and product development and technological innovations around probiotics have been growing rapidly [165]. However, probiotic strains are generally characterized by low heat resistance, instability, and possible intrinsic virulence factors of the strains and the development of drug resistance, which have limited their development and application in food and medicine [166]. The concept of postbiotics was first defined in 2013 by the Spanish scholar Tsilingiri as “probiotic-derived metabolites that produce beneficial effects on the host in a direct or indirect manner” [167]. The definition of postbiotics has evolved over time as research into postbiotics has progressed, and in 2021, the International Society for the Science of Probiotics and Prebiotics (ISAPP) defined postbiotics as “inanimate microorganisms and/or their constituents capable of delivering health benefits to their hosts” [11]. According to this definition, postbiotic elements include inactivated microorganisms, extracellular polysaccharides, cell-free supernatants, and SCFAs [168]. Table 2 outlines the categorization of postbiotics and some of their disease-related advantages.

The presence of pathogenic bacteria and toxic metabolites and a reduction in probiotics will break the balance of normal GM, leading to GM dysbiosis and gut metabolite disorders, which will make the host susceptible to infection with a variety of infectious and non-infectious diseases [13,169]. Postbiotic supplementation restores the composition and structure of the GM and promotes gut health. An increase in pathogens in the gut can lead to compromised gut health. It has been demonstrated that postbiotics compete with pathogens for adhesion to gut mucosa and epithelium [170]. Cell-free supernatant is one of the main active ingredients of postbiotics, which contains microbial metabolites and residual nutrients from the culture medium used, mainly including organic acids, fatty acids and proteins, with antibacterial, antioxidant, and anti-inflammatory properties [171]. The results of in vivo experiments in neonatal rats by He et al. [172] found that LGG culture supernatant pretreatment reduced bacterial colonization, translocation, and dissemination in the gut tract and significantly reduced the susceptibility of neonatal rats to oral *E. coli* K1 infection. Warda et al. [173] found that heat-inactivated *Lactobacillus fermentum* and *Lactobacillus delbrueckii* subsp. *delbrueckii* reduced the abundance of harmful bacteria such as *Clostridium* and *Turicibacter*, which ameliorated gut pathologic damage in colitis-affected mice and helped them regain their health. Several studies have shown that the addition of postbiotics to broiler diets enhances the immune response and promotes gut health by increasing the production of *Lactobacillus*, lowering *Enterobacteriaceae* and fecal pH, and improving gut villi [174,175,176].

Notably, SCFAs are considered to be an important postbiotic active ingredient that is a key end product of gut microbial activity [20]. Most gut diseases lead to inflammation, which triggers an immunological response. The most common SCFAs include acetic, propionic, and butyric acids, which can form their corresponding fatty acid salts (acetate, propionate, and butyrate) in the gut. The major SCFAs have been shown to promote the absorption of colonic salts and fluids, as well as the proliferation of colonic cells [168,177]. Butyrate is considered to be one of the most important sources of energy for enterocytes because of its contribution to the renewal of gut epithelial cells, while butyrate also exhibits immunosuppressive effects [178]. Pandey et al. [179] found that butyrate was able to reduce the production of pro-inflammatory cytokines including IL-8, IL-6, IL-12, and TNF-α and increase the production of anti-inflammatory cytokines to maintain the health of gut epithelium. Thananimit et al. [180] showed that *Lactobacillus paracasei* SD1, *Lactobacillus rhamnosus* SD11, and *Lactobacillus rhamnosus* GG exhibited high butyric acid production, especially in the mixed group of *Lactobacillus paracasei* SD1 and *Lactobacillus rhamnosus* SD11. The SCFAs in this group were able to inhibit the growth of colon cancer-associated pathogens, such as *Clostridium nucleolaris* and *Porphyromonas gingivalis*, and exerted improved colon cancer and safeguarded gut health.

In recent years, the use of postbiotics in animal production has received increasing attention. The addition of *Lactobacillus plantarum* (strains 22F, 25F, etc.) and *Pediococcus acidilactici* 72N dried at high temperatures to piglet diets improves feed conversion, increases fecal *Lactobacillus* counts, and reduces the number of potentially harmful bacteria such as *Enterobacter* [181]. It has also been shown that the addition of metabolites of *Lactobacillus plantarum* (strains TL1, RG11, RG14, RS5, RI11, etc.) to diets reduces the incidence of diarrhea in piglets after weaning and increases the concentration of *Lactobacillus* and SCFAs in the feces [182]. The use of postbiotics in poultry has also been studied. Supplementation of broiler with heat-killed *Enterococcus faecalis* strain EC-12 increases total IgA in cecal digestive fluid and total IgG in serum and reduces *vancomycin-resistant enterococci* (VRE) colonization of the intestine, suggesting that this post-organism stimulates the gut immune system and enhances the immune response to a VRE challenge to accelerate defecation in broiler [183]. Johnson et al.’s [184] study in broilers showed that a post-product containing a mixture of *Pediococcus acidilactici*, *Lactobacillus reuteri*, *Enterococcus faecium*, and *Lactobacillus acidophilus* alleviated the inflammatory response to *Clostridium difficile*. In addition, the addition of *Lactobacillus plantarum* (RI11, RS5, UL4, and other strains) supernatant to the diet reduced *Salmonella*, increased *Bifidobacterium* and *Lactobacillus*, and increased plasma IgM and IgG levels in the gut of broilers, which ensured the health of the gut and improved the heat stress of broilers [185]. Hylak^®^ Forte (Ratiopharm/Merckle GmbH, Ulm, Germany) is a postbiotic preparation containing metabolites (such as SCFAs, amino acids, and vitamins) from *E. coli* DSM 4087, *Streptococcus faecalis* DSM 4086, *Lactobacillus acidophilus* DS 414, and *Helactobacillus* DS 4183. It has been used to treat bacterial imbalances in the gastrointestinal to protect gut health and alleviate symptoms associated with it (such as flatulence, diarrhea, and constipation) [178]. However, it is also crucial to note that careful consideration must be given to the timing and dosage when administering postbiotics and their formulations in young or immunocompromised animals. Postbiotics typically activate the host’s immune system, and precautions should be taken to avoid excessive immune activation, which may lead to organismal damage. Additionally, attention should be paid to the fact that antimicrobial components produced by postbiotics may non-specifically inhibit commensal bacteria, thereby disrupting the balance of the host’s intestinal microecology.

**Table 2:** The classification of postbiotics and their beneficial effects on diseases.

The Active Ingredients of the Postbiotics	Source Strain	Research Object	Intervention Time	Outcomes	References
Cell-Free Supernatants	*Lactobacillus plantarum* TL1, RG11, RG14, RS5, and RI11	Wean piglets	5 weeks	↑ *Lactobacillus* and SCFAs	[182]
	*Lactobacillus plantarum* RI11, RS5, and UL4	One-day-old fifty-two Cobb 500 male chicks	21 days	↓ *Salmonella;* ↑ *Bifidobacterium* and *Lactobacillus*, IgM and IgG	[186]
	*Lactobacillus plantarum* RG14	Newly weaned lambs	60 days	↑ IL-6, TJP-1, CLDN-1, and CLDN-4; ↓ IL-1β, IL-10, and TNF	[187]
	*Saccharomyces cerevisiae* PTCC 5269	SW480 colon cancer cells	48 h	↓ *Listeria monocytogenes*, *Streptococcus mutans*, *Salmonella typhi*, and *E. coli*	[188]
Exopolysaccharides	*Lactobacillus plantarum* NCU116	Eight-week-old C57BL/6 male mice	7 days	↑ Claudin-1, Occludin, and ZO-1 ↓ TNF-α, IFN-γ, and IL-6	[189]
	*Lactobacillus delbrueckii* subsp. *delbrueckii* TUA4408L	Porcine gut epitheliocytes	48 h	↓ IL-6, IL-8, and MCP-1	[190]
Inactivated cells	*Enterococcus faecalis* strain EC-12	Newly hatched chicks	15 days	↑ Total IgA and total IgG ↓ *Vancomycin-resistant enterococci* (VRE) colonization of the gut	[182]
	*Streptococcus salivarius* M18	Human CRC epithelial cell lines HCT-116 and SW-480 (ATCC)	Indicated period of time	↓ *Pseudomonas aeruginosa*, *Klebsiella pneumonia*	[191]
	*Pichia kudriavzevii* FZ12	Weaned piglets	3, 6, and 12 days	↑ Beneficial bacteria, promoted growth performance, improved gut health performance	[192]
	*Bacillus subtilis* ACCC 11025	480-day-old broilers	21 and 42 days	↓ *Salmonella;* ↑ *Lactobacillus* bacteria	[193]
Bacteriocin	*Lactobacillus gasseri*	Different bacteria	/	↓ *Listeria monocytogenes*, *Bacillus cereus*, and *Staphylococcus aureus*	[194]
Short-chain fatty acid	*Roseburia intestinalis*	Eight-week-old GF male ApoE^−/−^ mice	18 weeks	↓ Lipopolysaccharide and TNF-α	[195]

Note: ↑ means promoting effect, ↓ means inhibiting effect.

## 6. Conclusions

With the continuous improvement of living standards, both humans and animals pay more and more attention to health. At the same time, gut health threats and diseases are developing more and more, and gut health has become a focus of attention for both humans and animal husbandry. As the largest microbial community in the organism, GM has gradually become widely studied as a target for improving gut health. Antibiotics are one of the greatest discoveries of the 20th century, and their application in animal husbandry has greatly improved the performance of animals and increased the economic benefits of the livestock industry. However, with the long-term use of antibiotics, their drawbacks have gradually become apparent, such as the development of resistance, significant toxic side effects, and high levels of residue. Therefore, the search for natural alternatives to antibiotics has become an important research direction in the livestock industry.

With the rapid development of next-generation sequencing (NGS) technology in the 21st century, various omics technologies, such as microbial genome sequencing, transcriptomics, and metagenomics, support us in further studying and understanding GM. Probiotics, prebiotics, synbiotics, and postbiotics (PPSP) have attracted much attention due to their wide sources, high safety, low likelihood of developing resistance, and ability to exert their beneficial effects through different pathways, leading to rapid development. In modern livestock and poultry farming, the development of encapsulation technology for PPSP, an important bioproduct for regulating gut health, is of critical significance. Effective encapsulation can protect active ingredients from adverse external factors such as gastric acid, bile, high temperatures, and humidity, ensuring that they remain viable upon reaching specific sites in the gut. This not only enhances the efficacy of the bioproduct but also improves its stability and controlled release characteristics, thereby facilitating precise interventions in gut health. The future development of probiotic encapsulation technology will increasingly focus on intelligence and personalization. On one hand, there is a need to develop smart encapsulation materials that can respond to changes in the gut environment, such as pH, temperature, and enzyme concentration, to achieve precise release of probiotics. On the other hand, personalized encapsulation strategies will be tailored according to the characteristics of different probiotic strains and their application requirements, maximizing their beneficial effects. Furthermore, with the continuous advancement of nanotechnology, nanoscale encapsulation materials are expected to further enhance the bioavailability and therapeutic efficacy of probiotics. In addition, CRISPR (Clustered Regularly Interspaced Short Palindromic Repeats), a gene editing tool that precisely targets specific locations in the microbial genome with Cas proteins (e.g., Cas9) and guide RNAs (gRNAs), has been applied to probiotics, known as CRISPR-engineered probiotics, addressing their inherent limitations. The application of this technology enables targeted gene editing while preserving native functions, thereby enhancing pathogen resistance, optimizing metabolic or immune regulation, and maximizing probiotic efficacy.

However, the application of PPSP in real environments still faces multiple challenges. First, the stability of PPSP is susceptible to environmental factors such as high temperatures, humidity, and mechanical stress, especially affecting the survival rate of probiotics. Although current embedding technologies can partially address this issue, their process complexity and high costs restrict large-scale application in animal husbandry. Additionally, results from in vitro experiments often struggle to fully replicate in vivo or field environment. For example, probiotics demonstrating excellent acid tolerance and adhesion in vitro may be inactivated within the complex gastrointestinal environment of animals due to pH fluctuations, digestive enzyme activity, or host immune responses. Thus, future research should systematically evaluate the stability and efficacy of in vitro-screened strains or compounds using animal models and further validate their applicability under large-scale farming conditions. Second, the production and addition costs of PPSPs are significantly higher than those of traditional antibiotics, posing challenges to the economic viability of the farming industry. Furthermore, considering the substantial variation in PPSP requirements across different animal species and growth stages, as well as existing regulatory barriers, many countries and regions have not yet established uniform standards and regulations for PPSP use, and the system for evaluating their safety and efficacy still requires improvement. Therefore, continuous efforts are needed to optimize technologies, reduce costs, and advocate for policy support to facilitate the industrialized and universal production of PPSP and promote its widespread adoption in animal husbandry.

Based on the above summary, PPSP can be used individually or in combination as green feed additives to directly or indirectly regulate the GM through mechanisms such as inhibiting the invasion of pathogenic bacteria, promoting the growth of beneficial bacteria, enhancing the intestinal barrier and triggering the immune effect, thereby promoting gut health. (Table 3 and Table 4 summarizes some of the roles, safety, challenges, and comparisons of PPSP). In the future, modern technologies such as molecular biology, genetic engineering, systems biology, omics, nanotechnology, and immunology will help us understand GM and discover and develop new PPSP to promote human and animal health, which will become the new trend in future explorations.

## Figures and Tables

**Table 1:** Probiotic strains used to regulate the gut microbiota.

Probiotic Strains	Research Object	Disease	Intervention Time	Therapeutic Outcome	References
*Lactobacillus helveticus*	HEp-2 and T84 epithelial cells	/	18 h	Decreased pathogen adherence and attaching-effacing lesions in addition to preserving the barrier function of monolayers.	[33]
*Lactobacillus rhamnosus* GG	Weaned pig	The monophasic variant Salmonella	7 days	Removed or reduced the residence of pathogenic bacteria, produce substances that can antagonize food-borne pathogenic bacteria, and also directly participate in the repair of damage to the intestinal mucosal barrier.	[34]
*Lactobacillus acidophilus* SW, *Lactobacillus fermentum* 33, *Lactobacillus plantarum* L05, and *Enterococcus faecium* TM39	Newly hatched Arbor Acres broiler chicks	*Salmonella*	3 days	Up-regulated the ratio of *Firmicutes/Bacteroidetes* and increased the proportion of genus of *Clostridiales*.	[35]
*Lactobacillus plantarum* JDFM LP11	Six female three-way crossbred piglets	/	4 weeks	Increased the population of lactic acid bacteria in feces and enhanced the development of villi in the small intestine.	[36]
*Lactobacillus*, *Lactococcus* and *Bifidobacterium*	One-day-old Arbor Acres broilers	*/*	42 days	Increased the relative abundance of *Bacteroidales*, *Rikenellaceae* and *Alistipes*, enhanced the cecal microbiota, and decreased the relative abundance of harmful microorganisms.	[37]
*Saccharomyces cerevisiae* var. *boulardii*	Eighteen-day-old pigs	*Enterotoxigenic E. coli* infection	18, 24, 42 and 52 days	Reduced the adhesion of *enterotoxigenic E. coli* to mesenteric lymph nodes.	[38]
*Saccharomyces cerevisiae* subsp. *boulardii* SB-CNCM I-1079	Wean piglets	*/*	37 days	Promoted the establishment of *Porphyromonadaceae* and *Ruminococcaceae* in the colon and influenced the intestinal and colonic microbiota.	[39]
*Lactobacillus acidopilus*	Eight-week-old male C57BL/6 mice	Inflammatory bowel disease	7 days	Suppressed IL-6, TNF-α, IL-1β, and IL-17; modulated the balance between Th17 and Treg cells	[40]
*Bifidobacterium infantis*	Eight-week-old SPF male SD rats	Nonalcoholic fatty liver disease	12 weeks	Improved gut microbiota structure and liver pathology; downregulated serum LPS and liver TLR4.	[41]
*Lactobacillus plantarum* KLDS1.0318, *Lactobacillus plantarum* KLDS1.0344, *Lactobacillus plantarum* KLDS1.0386 and *Lactobacillus plantarum* WCSF1	Six-week-old, SPF female 70 BALB/c mice	LPS-induced intestinal injury	15 days	Decreased TNF-α, IL-6 and IL-12 levels, increased the number of CD4^+^ T cells and IgA plasma cells and the expression Claudin1 and Occludin, and increased the relative abundance of *Lactobacillus*, *Lachnoclostridium*, and *Desulfovibrio.*	[42]
*Lactobacillus acidophilus*, *Lactobacillus reuteri* and *Lactobacillus salivarius*	Newly hatched female commercial broiler chicks	/	4, 5, and 6 weeks	Enhanced the antibody levels and cell-mediated immunity.	[43]
*Bifidobacterium dentis*	Eight–sixteen-week-old GF female mice	/	17 days	Limited the interaction of harmful microbiota with epithelial cells in the intestinal lumen and inhibited the growth of *E. coli*, *Clostridium difficile*, *Salmonella*, *Helicobacter pylori*, and *Listeria*.	[44]
*Bifidobacterium longum DD98*	Six–eight-week-old C57BL/6 male mice	Ulcerative colitis	14 days	Improved the diversity of gut microbiota, promoted the proliferation of *Trichoderma*, *Lactobacillus*, and *Prevotella*, enriched the intestinal population of *Bacteroides* and *Clostridium leptum*, and enhanced butyric acid metabolism.	[45]
*Bifidobacterium longum* subsp. *Infantis* LR655210.1	Four-week-old male C57BL/6 mice	*Enterotoxigenic E. coli* K88-induced diarrhea	14 days	Recovered weight and colon length to a certain extent and down-regulated the levels of IL-6 and TNF-α.	[46]
*Bifidobacterium shortum MCC-117*	Porcine intestinal epithelial (PIE) cells	*Enterotoxigenic E. coli*-induced inflammation	48 h	Stimulated the expression of TLR-2 and COX-2 in the ileal epithelium and blocked cytokine-induced apoptosis.	[47]
*Lactobacillus suspension* or *Lactobacillus plantarum*	Layer	*C. jejuni-* and *Saccharomyces Enteritidis*-induced infections	4 days	Enhanced gut colonization resistance to *C. jejuni* and upregulated IL-6, IL-10, and TLR4 in ileum.	[48]

**Table 3:** The role, safety, and challenges of PPSP in the article are summarized.

Category	Efficacy	Safety	Key Application Challenges	Recommended Species
Probiotics (e.g., *Lactobacillus*, *Bifidobacterium*, *Saccharomyces*)	1. Modulate gut microbiota 2. Enhance gut barrier 3. Boost immunity 4. Species/strain-specific effects	Generally recognized as safe (GRAS)	1. Low survival during feed processing/storage 2. Variable colonization in the gut 3. Potential antibiotic resistance gene transfer	Pigs, poultry, calves
Prebiotics (e.g., FOS, IMO, GOS, inulin)	1. Selective growth of beneficial bacteria (e.g., *Bifidobacterium*) 2. SCFAs production 3. Immune modulation	Safe, non-digestible	1. Dose-dependent effects 2. Fermentation may cause bloating 3. Variable efficacy across diets	Poultry, weaned piglets
Synbiotics (probiotic + prebiotic)	1. Enhanced probiotic survival 2. Synergistic microbiota modulation 3. Improved growth performance	Safe if components are GRAS	1. Optimal pairing required 2. Higher cost 3. Stability during processing	Broilers, sows, piglets
Postbiotics (e.g., inactivated cells, metabolites, SCFAs)	1. Stable under processing 2. Anti-inflammatory/antibacterial effects 3. No live bacteria risks	High safety (no viability concerns)	1. Limited long-term studies 2. Production scalability 3. Regulatory ambiguity	Poultry, piglets

**Table 4:** Comparison of the mechanism of PPSP in the article.

Category		Main Mechanism	Key Metabolites/Components
Probiotics		1. Competitively inhibit pathogen colonization 2. Enhance intestinal barrier (e.g., up-regulate occludin) 3. Modulate immunity (e.g., promote sIgA secretion)	Lactic acid, acetic acid, bacteriocins
Probiotics		1. Selectively promote proliferation of beneficial bacteria 2. Fermentation to produce SCFAs 3. Reduce intestinal pH to inhibit pathogens	Short-chain fatty acids (SCFAs), oligosaccharides (e.g., FOS, GOS)
Synergistic		1. Probiotics and prebiotics synergy 2. Increased probiotic survival rate 3. Enhanced metabolite production	SCFAs, vitamins
Postbiotics	Inactivated cells	1. Physical adsorption of pathogens (cell wall binding) 2. Immunomodulation (TLR2/4 activation) 3. Competitive occupancy	Peptidoglycan, lipophosphatidic acid
	Short-chain fatty acids	1. Energy supply (colonic cell butyric acid utilization) 2. pH lowering inhibition of pathogens 3. Regulate Treg differentiation	Acetic acid, propionate, butyrate
	Antimicrobial peptides	1. Directly cleave pathogen cell membranes 2. Inhibit biofilm formation Bacteriocins	Bacteriocin, defensins
	Exopolysaccharides	1. Physical barriers to protect probiotics 2. Induction of immune tolerance (e.g., IL-10 upregulation) 3. Binding of heavy metals/toxins	β-glucan, hyaluronic acid
	Extracellular vesicles	1. Delivery of nucleic acids/proteins to regulate host cells 2. Cross-species signaling	miRNA, functional enzymes

## Data Availability

Not applicable.

## Abbreviations

GM	Gut microbiota
PPSP	Probiotics, Prebiotics, Synbiotics, and Postbiotics
FAO	Food and Agriculture Organization
WHO	World Health Organization
GIT	Gastrointestinal tract
*E. coli*	*Escherichia coli*
LGG	*Lactobacillus rhamnosus* GG
GRAS	Generally recognized as safe
TLR-2	Toll-like receptor 2
COX-2	Cyclooxygenase-2
ISAPP	International Society for the Science of Probiotics and Prebiotics
FOS	Fructooligosaccharides
XOS	Xylo-oligosaccharide
IMO	Isomalto-oligosaccharides
GOS	Galactooligosaccharides
APEC	Avian pathogenic E. coli
UC	Ulcerative colitis
DP	Degrees of polymerization
gRNAs	Guide RNAs
CRISPR	Clustered Regularly Interspaced Short Palindromic Repeats
NGS	Next-generation sequencing
SCFAs	Short-chain fatty acids
